# Sympathetic skin response in multiple sclerosis: a meta-analysis of case-control studies

**DOI:** 10.1007/s10072-017-3111-6

**Published:** 2017-09-29

**Authors:** Nicolò Margaritella, Laura Mendozzi, Massimo Garegnani, Elisabetta Gilardi, Raffaello Nemni, Luigi Pugnetti

**Affiliations:** 1 Laboratory of Clinical Neurophysiology, Scientific Institute (IRCCS) S. Maria Nascente, don C. Gnocchi Foundation, via Capecelatro 66, 20148 Milan, Italy; 2 Multiple Sclerosis Rehabilitation Unit, Scientific Institute (IRCCS) S. Maria Nascente, don C. Gnocchi Foundation, Milan, Italy; 3 Neurological Rehabilitation Unit, Scientific Institute (IRCCS) S. Maria Nascente, don C. Gnocchi Foundation, Milan, Italy

**Keywords:** Sympathetic skin response, Multiple sclerosis, Meta-analysis, Case-control, Evoked potentials, Autonomic nervous system

## Abstract

The usefulness of sympathetic skin responses (SSR) in multiple sclerosis (MS) has been advocated by several studies in the last 20 years; however, due to a great heterogeneity of findings, a comprehensive meta-analysis of case-control studies is in order to pinpoint consistencies and investigate the causes of discrepancies. We searched MEDLINE, EMBASE and Cochrane databases for case-control studies comparing SSR absence frequency and latency between patients with MS and healthy controls. Thirteen eligible studies including 415 MS patients and 331 healthy controls were identified. The pooled analysis showed that SSR can be always obtained in healthy controls while 34% of patients had absent SSRs in at least one limb (95% CI 22–47%; *p* < 0.0001) but with considerable heterogeneity across studies (*I* ^2^ = 90.3%). Patients’ age explained 22% of the overall variability and positive correlations were found with Expanded Disability Status Scale and disease duration. The pooled mean difference of SSR latency showed a significant increase in patients on both upper (193 ms; 95% CI 120–270 ms) and lower (350 ms; 95% CI 190–510 ms) extremities. We tested the discriminatory value of SSR latency thresholds defined as the 95% confidence interval (CI) upper bound of the healthy controls, and validated the results on a new dataset. The lower limb threshold of 1.964 s produces the best results in terms of sensitivity 0.86, specificity 0.67, positive predicted value 0.75 and negative predicted value 0.80. Despite a considerable heterogeneity of findings, there is evidence that SSR is a useful tool in MS.

## Introduction

The sympathetic skin response (SSR) is a simple, reproducible and non-invasive test based on the modification of the skin potential elicited most frequently by electrical stimulation of peripheral nerves. It is one of the methods to assess the autonomic nervous system (ANS) as it involves peripheral pre- and post-ganglionic sympathetic sudomotor fibres as well as central structures such as the posterior hypothalamus, upper brain-stem reticular formation and spinal cord [1].

Multiple sclerosis (MS) is an inflammatory demyelinating disease with a scattered involvement of the central nervous system that can also affect the ANS [2]. The ANS can be impaired in up to 90% of the subjects with multiple sclerosis (MS) [3]; even if the involvement of the ANS impacts the quality of life of MS patients [4], it is often overlooked due to insufficient clinical follow-up and assessment procedures [3]. The analysis of SSR in MS case-control studies started around 1990 and spanned a 20 years’ period during which several small studies have been published advocating the use of SSR in SM, in spite of a substantial heterogeneity in their methods and results.

Here, we set out to perform a comprehensive meta-analysis of case-control studies that have investigated the association of SSR with MS. We also ran a simulation study exploiting the information of previous studies to evaluate whether a suitable threshold of SSR latency may discriminate MS patients from healthy controls, and validated the results on a new dataset collected by our laboratory. Finally, we attempted to understand the variability of results across studies testing covariates which may represent meaningful sources of heterogeneity.

## Methods

Eligible articles reporting the prevalence of missing SSR among patients with MS and healthy controls were identified by searching MEDLINE, EMBASE and the Cochrane Database of Systematic Reviews databases. Studies published up to December 2015 were searched using the following combination of search strings: (‘sympathetic skin response OR SSR’) AND ‘multiple sclerosis’. No language restrictions were imposed. Reference lists of all articles that met the eligibility criteria were examined to identify studies that may have been missed by the database search.

Studies were eligible if they met the following criteria: (i) report of original data in a peer-reviewed publication, (ii) evaluation of patients with MS in a case-control setting against a healthy control group, (iii) use of electrical stimulation of the limbs to evoke the SSR and (iii) report of the number of patients and controls with/without the SSR response in at least one limb and, if available, the SSR latency in the two groups. Subjects with other neurological disorders in control groups were excluded from the analysis. Duplicate publications were also excluded. In the event that the full-text version of a study did not report clearly the number of patients and controls with missing SSR response as well as the SSR latency mean (SD) for both groups, the corresponding author was asked to provide the missing data in order to maximise the amount of summarized data. We removed studies whose authors did not respond. The following data were collected (when available): journal name, year of publication, number of missing SSR responses in MS patients and controls, age, sex, type of MS, disease duration, Expanded Disability Status Scale (EDSS), SSR stimulation site, SSR intensity and duration, inter-stimulus interval, skin temperature and SSR latency.

Because none of the controls had missing SSR responses, we used pooled proportions to evaluate the overall prevalence of missing responses within the MS patient group. The latency mean difference between patients and healthy controls was computed from studies reporting SSR latencies. The fixed-effects model (Mantel-Haenszel) or random-effects model (DerSimonian Laird) were used to compute the pooled estimates. Heterogeneity between studies was assessed by the Cochran *Q* and *I* ^2^ statistics. When statistically significant heterogeneity was detected (Cochran Q *p* value < 0.1), random-effect models were employed. We referred to the *I* ^2^ statistics for the qualitative interpretation of heterogeneity; values greater than 50% are usually considered as indicative of substantial heterogeneity, while values greater than 75% represent considerable heterogeneity [5]. When a substantial or a greater amount of heterogeneity was discovered, possible covariates were tested using mixed-effects meta-regression models when possible, or explorative correlations in the presence of missing data [6]. Publication bias was assessed on the overall analysis; to maximise the power of the test Egger’s statistical test was performed.

In addition to the meta-analysis study, we tested the 95% confidence interval of the controls’ pooled mean latency for both superior and inferior limbs as diagnostic thresholds. We simulated 5000 data from each study enrolled in the meta-analysis using their SSR mean and standard deviation (SD) from both upper and lower limbs and assuming normality; subsequently, we computed the overall diagnostic odds ratio (DOR) and the area under the curve (AUC) statistics for superior and inferior limbs for each repetition and extracted mean and 95% empirical CI.

We recorded the SSR from SM patients and healthy controls to validate our results using the following experimental setting: we stimulated each limb with a single pulse of 10–20-mA intensity and 0.2-ms duration and recorded the first elicited ipsilateral response among 3 to 5 stimuli delivered at more than 60-s interval each. We did not attempt to control limb temperature through direct warm up of the extremities but used a wool blanket to maintain the limb temperature during the entire examination period. All recordings were conducted using XLTEK® Protector 16-channel averager and statistical analyses using R version 3.2.3 [7] with packages metafor [6] and mada [8].

## Results

**Fig. 1:**
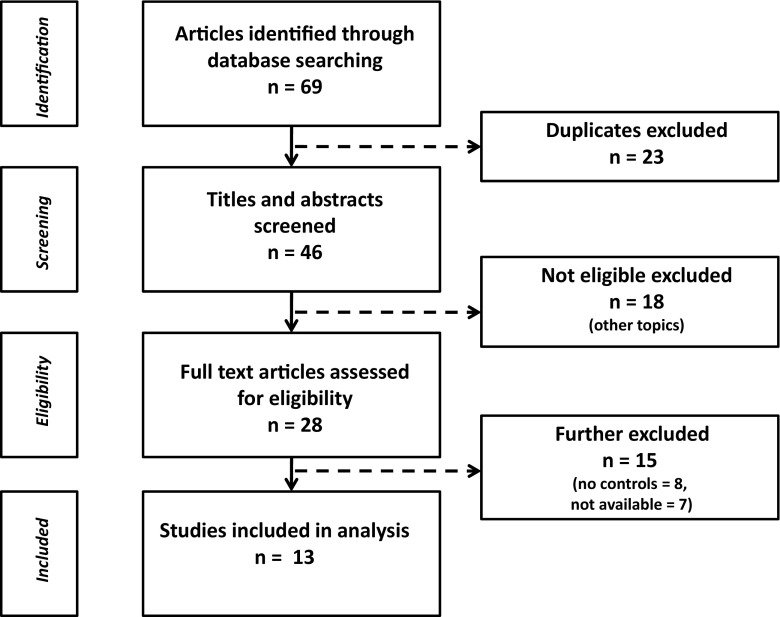
Flow chart showing the selection of eligible studies

In the first step of the database search, we identified 69 studies; 16 were from the EMBASE database. After the exclusion of 23 duplicate studies, the remaining 46 were screened for eligibility. Out of 28 potentially eligible studies, 15 were excluded either because of a missing control group (8 studies) or because of missing information (7 studies) which could not be recovered but in one case, either because the studies were too old and the authors could not retrieve their data or because they did not respond to our request. Data were extracted from the remaining 13 studies (Table 1 and Fig. 1).

**Table 1:** Demographic characteristics of MS patients and healthy controls enrolled in the case-control studies which were included in the present meta-analysis. Some data were not available (n/a)

Study	Year	Country	EDSS	Duration	Total	MS	Women	Control		
						age		Total	Age	Women
Karaszewski et al.	1990	USA	n.a.	n.a.	23	n/a	n/a	24	n/a	n/a
Yokota et al.	1991	Japan	n.a.	n.a.	28	44	22	50	44.6	n/a.
Gutrecht et al.	1993	USA	3.2	n.a.	29	42(11)	21	26	38(12)	21
Elie et al.	1994	France	n.a.	n.a.	70	37(11)	45	35	35(8)	24
Drory et al.	1995	Israel	1.8	8.5	60	39	35	30	38	17
Matsunaga et al.	1995	Japan	n.a.	n.a.	10	n/a	n/a	12	32	n/a
Zakrzewska-Pniewska et al.	1996	Poland	4.2(1)	9.8(8)	25	38(7)	13	26	38(11)	13
Alavian-Ghavanini et al.	1999	Iran	n.a.	3.8	30	31	20	14	n/a	n/a.
Gunal et al.	2001	Turkey	1.8(1)	8(6)	22	37(8)	20	22	38(8)	19
Nazliel et al.	2002	Turkey	2.1(2)	3.7	21	38(11)	13	25	37(10)	13
Secil et al.	2007	Turkey	2.3(0.2)	4.8(0.6)	40	36	40	20	46	20
Saari et al.	2008	Finland	4.2(3)	6.1(5)	27	38(8)	14	27	40(9)	14
Aghamollaii et al.	2011	Iran	1.86	3.1(3)	30	32(9)	15	20	30(8)	11
Present study^a^	2016	Italy	1.36(1)	10.2(7)	22	36(6)	13	18	34(8)	10

^a^The data we collected were not used in the meta-analysis but to validate the results of the simulation study

There were three studies from Turkey, two from Iran, two from Japan and two from the USA. The remaining studies were conducted in Finland, France, Israel and Poland. Two studies were published on Journal of Neurological Sciences and two on Acta Neurologica Scandinavica. The remaining studies were published on Annals of Neurology, Brain, Multiple Sclerosis, Clinical Neurophysiology, Muscle&Nerve, European Neurology, Neurologia i Neurochirurgia Polska, Electromyography and Clinical Neurophysiology.

The total number of MS patients and healthy controls included in the final meta-analysis was 415 and 331, respectively. The SSR mean latency and standard deviation (SD) were not reported in four studies [9–12]. We derived the SD in one study [13] from the reported range, assuming normality. Demographic characteristics of patients with MS and healthy controls are reported in Table 1. SSR recording methods are reported in Table 2; to avoid habituation, the inter-stimulus interval was ≥ 30 s in three studies [10, 12, 14], ≥ 45 s in one study [2], ≥ 60 s in five studies [3, 11, 15–17], ≥ 90 s in one study [13] and random in two studies [18, 19]. Six studies kept the skin temperature over 31–32 °C [3, 9–11, 13, 17] and one study over 34 °C [14] while the other six studies did not report whether they controlled the skin temperature and, if they did, omitted to report the threshold value.

**Table 2:** SSR recording features for the studies analysed. Some data were not available (n/a). *RR* relapsing-remitting, *SP* secondary progressive, *PP* primary progressive, *CIS* clinically isolated syndrome, *R* right, *L* left, *MT* motor threshold

Study	MS course	Stimulus site	Intensity (mA)	Duration (ms)	Latency recorded	Absence definition
Karaszewski et al.	Progressive	R foot	n/a	0.2	n/a	n/a
Yokota et al.	Definite MS	Supraorb.	10–30	0.2	Shortest of 10	n/a
Gutrecht et al.	Definite MS	Median	6–12	0.2	Average of 4	n/a
Elie et al.	RR, SP, PP	R median	MT	0.5	n/a	n/a
Drory et al.	RR, SP	R median	MT	0.1	First elicited	After 4 stimuli of increasing intensity
Matsunaga et al.	n/a	R median	20	0.2	Shortest of 8	After a second stimulus of 30 mA
Zakrzewska-Pniewska et al.	RR, SP	R, L median	MT	0.5	n/a	n/a
Alavian-Ghavanini et al.	Definite MS	Median	20–60	0.1	Average of 3 to 5	n/a
Gunal et al.	RR	Median and tibial	70—median 100—tibial	0.1	n/a	After 5 stimuli
Nazliel et al.	Definite MS	Median and tibial	6–20	0.2	First elicited	After 10 stimuli of increasing intensity
Secil et al.	RR, SP, PP	R median	70	0.5	Average of 5	After 10–15 stimuli
Saari et al.	RR, SP	Median	20 to 30% > MT	0.5	Largest amplitude	After 4 stimuli
Aghamollaii et al.	RR, SP, PP, CIS	Median and tibial	> 10	0.2	n/a	After 10 stimuli of increasing intensity
Present study^a^	RR	Median and tibial	10–20	0.2	First elicited	After 3 to 5 stimuli

^a^The data we collected were not used in the meta-analysis but to validate the results of the simulation study

**Fig. 2:**
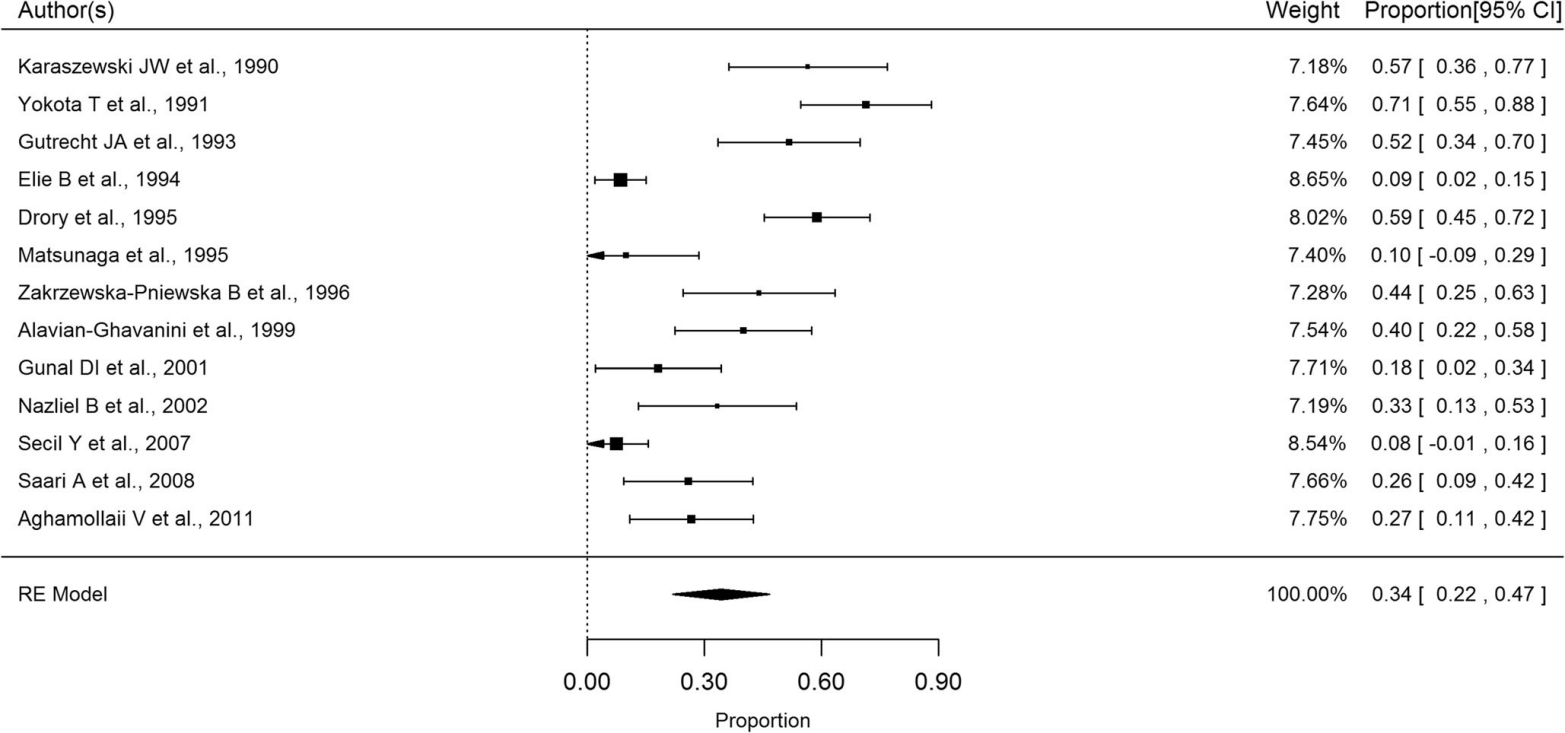
Forest plot describing the overall proportion of MS patients with at least one SSR absent response

None of the healthy controls had a missing SSR response. The proportion of MS patients with a missing SSR response varies widely across studies (Fig. 2) ranging from 71% (95% CI 55–88%) in the study of Yokota et al. (1991) [10] to 8% (95% CI 0–16%) in the more recent work of Secil et al. (2007) [19].

The pooled analysis showed that the proportion of MS patients with an absent SSR response is significantly different from zero (34%; 95% CI 22–47%; *p* < 0.0001) with considerable heterogeneity across studies (*I* ^2^ = 90.3%; *Q* = 123, *p* < 0.0001). Patients’ age explained 22% of the total heterogeneity indicating that, for instance, a 5-year increase in mean age adds another 17% to the total proportion of MS patients with an absent SSR response. Moreover, we found disease duration to be moderately correlated with the proportion of MS patients with an absent SSR response (*r* = 0.35) while the EDSS was weakly correlated (*r* = 0.25). No publication bias was evident (Egger’s test *p* = 0.68).

**Fig. 3:**
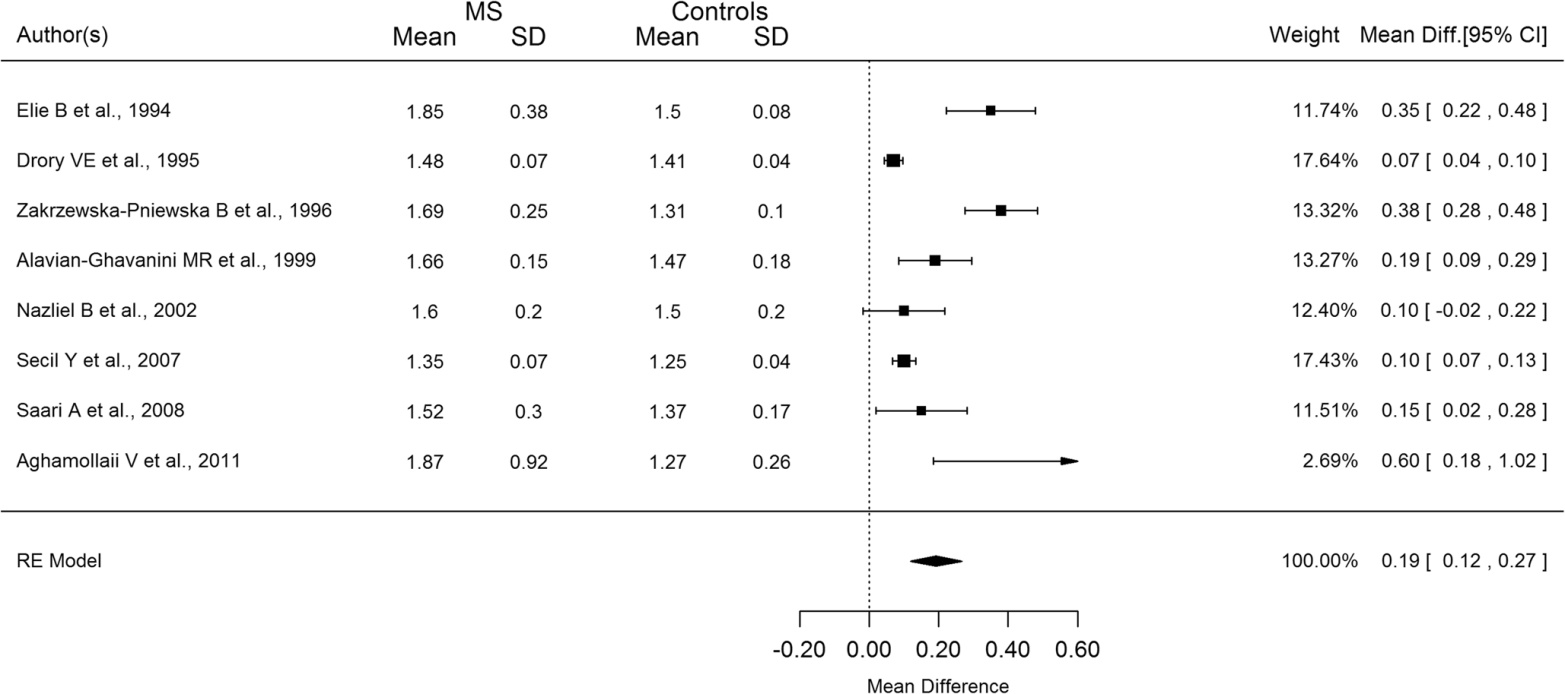
Forest plot describing the mean difference between MS patients and controls’ SSR upper limb latency

**Fig. 4:**
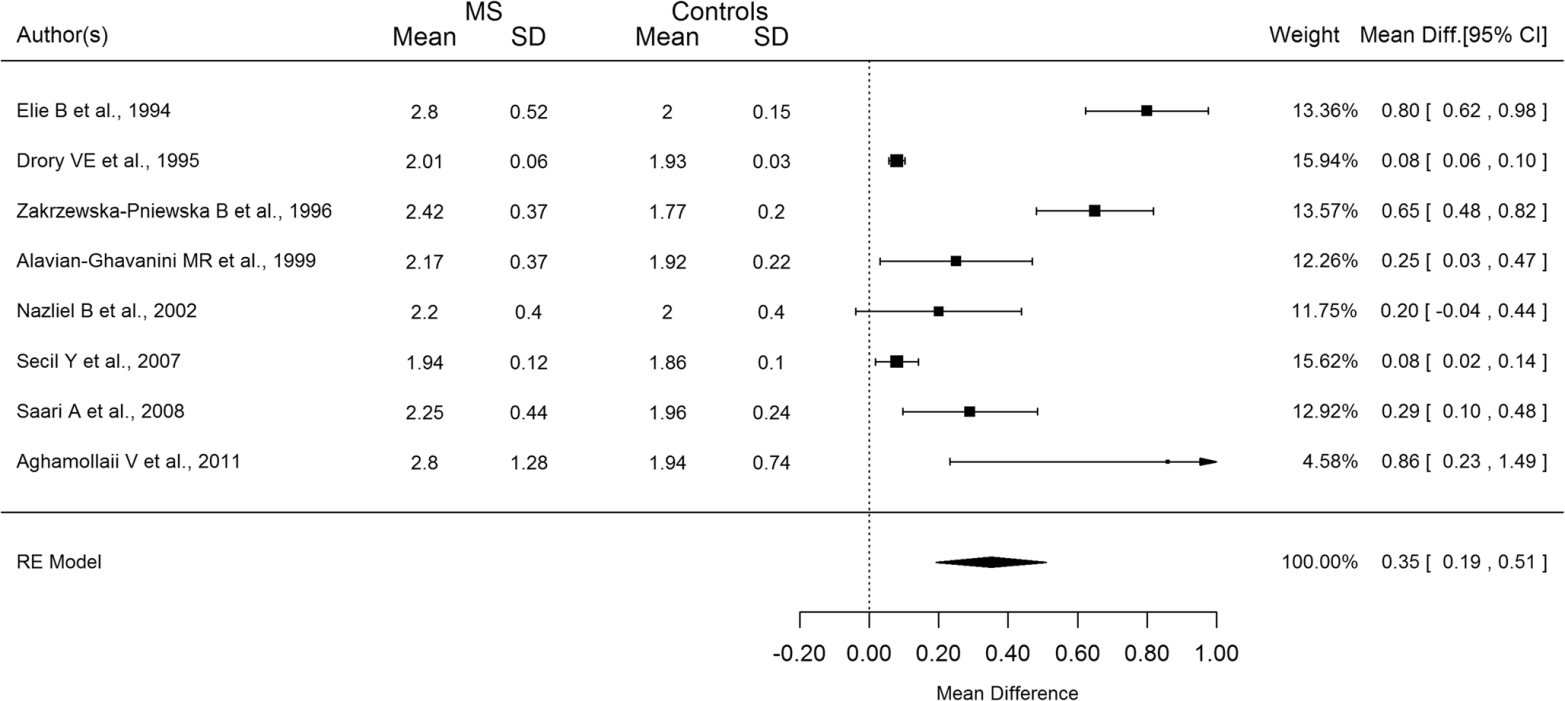
Forest plot describing the mean difference between MS patients and controls’ SSR lower limb latency

The mean difference in the SSR latency between MS patients and healthy controls varies greatly among studies for both upper and lower limbs (Figs. 3 and 4). The pooled analysis in Figs. 3 and 4 showed that the mean difference was significantly different from zero in the upper limbs (193 ms; 95% CI 120–270 ms) and in the lower limbs as well (350 ms; 95% CI 190–510 ms).

Considerable heterogeneity was apparent in both arms and legs (*I* ^2^ = 87.5 and 94%, respectively). The difference in mean latencies across studies was weakly correlated with EDSS and disease duration at all limbs; however, all the correlations increased (EDSS upper limbs: *r* = 0.77; EDSS lower limbs: *r* = 0.81; disease duration upper limbs: *r* = 0.52; disease duration lower limb: *r* = 0.53) after removing just one study [3].

The mean SSR latency of the healthy controls’ upper limbs was 1385 ms (95% CI 1308–1462 ms) and 1915 m (95% CI 1866–1964 ms) in the lower limbs. The results of the simulation study using the upper 95% CI of the healthy controls as a threshold showed that the diagnostic odds ratio (DOR) for the upper limbs was 5.81 (95% CI 3.80–8.66) and 6.53 (95% CI 4.1–10.1) for the lower limbs.

The area under the curve was 0.77 (95% CI 0.71–0.83) for the upper limbs and 0.81 (95% CI 0.74–0.88) for the lower limbs. The validation on our dataset showed a not significant DOR for the upper limbs (2.68; 95% CI 0.73–9.8) and a significant DOR for the lower limbs (12; 95% CI 2.5–57.5). The SSR latency threshold of the lower limbs showed a good discrimination ability with a sensitivity of 0.86 (95% CI 0.64–0.95), a specificity of 0.67 (95% CI 0.44–0.84), a positive predicted value (PPV) of 0.75 (95% CI 0.58–0.92) and a negative predicted value (NPV) of 0.80 (95% CI 0.60–1).

## Discussion

To our knowledge, this is the first meta-analysis of case-control studies to summarize the role of the sympathetic skin response (SSR) in multiple sclerosis (MS) patients. The pooled result of our study showed that one-third of MS patients has no SSR response at least at one limb; on the other hand, none of the 331 healthy controls in the studies considered had a missing SSR. These findings suggest that, although the presence of SSR responses at all limbs cannot rule out the disease, an absent SSR response should be considered a warning sign of a pathological condition affecting the central or peripheral sudomotor subsystem of the ANS [1].

Moreover, an absent or altered SSR in the early phases of MS may carry additional information that standard clinical measures, such as the Expanded Disability Status Scale (EDSS), cannot capture [18]. In addition, SSR testing may also provide further information when performed alongside more popular MS neurophysiological tests, such as the evoked potentials (EPs). This was suggested by Aghamollai et al. [3], who reported abnormal or absent SSR also in MS patients without EP anomalies. We found supporting evidence in our validation dataset, where one out of 5 patients with an absent SSR had an EDSS of 1.5 and multimodal sensory evoked potentials within normal limits.

The pooled analysis of the SSR latency mean difference showed a significantly longer latency in the MS patients, particularly in the lower limbs. The latency of the SSR was longer for the MS patients compared to that for the controls in all the studies included in the analysis. This argues against the uselessness of SSR latency measurement suggested by some studies [4, 18]. Furthermore, we showed that the 95% confidence interval (CI) upper limit of SSR latency of the healthy controls could be a suitable clinical threshold to discriminate MS patients from healthy subjects. The validation study on our dataset suggested that the lower limbs threshold (1964 ms) provides better discrimination. The use of a SSR latency threshold to discriminate MS patients from healthy controls was also tested by Aghamollai et al. [3] and Alavian-Ghavanini et al. [14], who reported similar good performances though they did not further validate their findings.

We found a considerable heterogeneity of both proportion and mean difference pooled analyses among case-control studies included in our meta-analysis. Patients’ age explained nearly 25% of the overall variability of the proportion of patients with absent SSR across studies. Although it is possible to consider age as a proxy of disease duration in the MS framework, it is worth mentioning that in a previous work [20] the absence of SSR was also related to ageing in healthy subjects. As a consequence, it is hard to interpret the meaning of the relationship between mean age and the proportion of absent SSR we found in the data.

In addition, the exploratory correlations between EDSS and both the proportion of absent SSR and SSR latency suggested that EDSS may help explain another part of the overall heterogeneity found in our meta-analysis. This agrees with some studies [3, 4] where both duration and severity of the disease were related to the involvement of the ANS.

Other sources of variability may be found in the different technical setting used to record the SSR. Habituation, body temperature, stimulus intensity and selection of the evoked response are important variables to consider [1, 21, 22].

Habituation in healthy subjects causes a progressive decrease of SSR amplitude that appears earlier if stimuli are given at a regular, predictable pace. To minimise habituation, a random stimulation with a frequency greater than one per minute is suggested [21]. In the present meta-analysis, we observed a variety of inter-stimulus intervals ranging from 30 to 90 s, making it possible for shorter intervals to inflate pathological findings.

Skin temperature was found to be negatively related to both SSR latency and amplitude in healthy subjects [21, 22], and the local warming of the extremities was shown to decrease SSR amplitude through depolarization of the sweat glands [21]. Not all the studies analysed here reported if and how skin temperature was measured and controlled during SSR recordings; failure to control this parameter could have also enhanced the heterogeneity of findings across studies.

It is known that the amplitude of the SSR depends on the stimulus strength [23] and that a weak stimulus may fail to induce an arousal response [1]. Due to the wide range of intensity and duration of the stimuli reported across studies, we cannot rule out the possibility that different combinations of intensity and stimulus duration have influenced the SSR measures to some extent.

Eventually, it must be stressed that even in studies of healthy subjects, there is still no consensus on the proper way to measure the skin responses and their mathematical processing [21]. The absolute amplitude value of the first response elicited was suggested to be representative of the peak amplitude and of the shortest latency; however, only two studies among those included in the meta-analysis employed this method. Three studies used averaging, even though it is affected by habituation and by shape variability across responses [24]. No consensus is also present on the definition of SSR absence. Some studies considered SSR absence if no response was elicited after 10 to 15 stimuli; however, in our experience, 3 to 5 stimuli per limb with a minimum inter-stimulus interval of 1 min increase testing time to an extent compatible with habituation and subjects’ discomfort.

Together with a considerable heterogeneity, missing data is another limitation in our study. Several controlling factors could not be properly tested in our meta-analysis due to missing data, the main source of which is the lack of a common approach in the collection of variables across the studies with some failing to report even standard demographic and clinical information.

Despite these limitations, the pooled results of this meta-analysis support SSR recording to detect ANS involvement in MS using the latency or the absence of response as a measure. Our findings suggest that SSRs yield complementary information to that of the EDSS and evoked potentials which could contribute to the understanding and monitoring of MS.
